# Avulsion fracture of the ischial tuberosity treated with the suture bridge technique: a case report

**DOI:** 10.1186/s12891-018-2377-z

**Published:** 2019-01-05

**Authors:** Tomonori Tetsunaga, Hirosuke Endo, Tomoko Tetsunaga, Kazuki Yamada, Takayuki Furumatsu, Toshifumi Ozaki

**Affiliations:** Department of Orthopaedics, Okayama University, 2-5-1 Shikata-cho, Kitaku, Okayama, 700-8558 Japan

**Keywords:** Injury, Ischial avulsion, Surgical repair, Suture bridge

## Abstract

**Background:**

In cases of avulsion fracture of the ischial tuberosity in which the bone fragments are substantially displaced, nonunion may cause pain in the ischial area. Various surgical procedures have been reported, but achieving sufficient fixation strength is difficult.

**Case presentation:**

We treated a 12-year-old male track-and-field athlete with avulsion fracture of the ischial tuberosity by suture anchor fixation using the suture bridge technique. The boy felt pain in the left gluteal area while running. Radiography showed a left avulsion fracture of the ischial tuberosity with approximately 20-mm displacement. Union was not achieved by conservative non-weight-bearing therapy, and muscle weakness persisted; therefore, surgery was performed. A subgluteal approach was taken via a longitudinal incision in the buttocks, and the avulsed fragment was fixed with five biodegradable suture anchors using the suture bridge technique.

**Conclusions:**

Although the majority of avulsion fractures of the ischial tuberosity can be treated conservatively, patients with excessive displacement require surgical treatment. The suture bridge technique provided secure fixation and enabled an early return to sports activities.

## Background

Pelvic apophyseal avulsion fracture is a category that includes a few types of avulsion fractures. Avulsion fractures of the anterior inferior iliac spine, anterior superior iliac spine, and iliac crest are comparatively common, but avulsion fractures of the ischial tuberosity are rarely described in the literature [1]. However, this fracture is frequently observed in athletes during growth spurts. The underlying mechanism involves damage to the vulnerable epiphyseal plate before epiphyseal arrest, caused by sudden and forceful eccentric contraction of the hamstrings and is attributed to sprinting or jumping. Bone union must be achieved, and range of motion (ROM) and muscle strength should be restored before full return to sports activities. However, patients with substantial displacement, sciatic nerve complications, or nonunion after conservative treatment require surgical treatment with adequate fixation. We report a case in which suture anchor fixation using the suture bridge technique was applied for the treatment of avulsion fracture of the ischial tuberosity.

## Case presentation

**Fig. 1:**
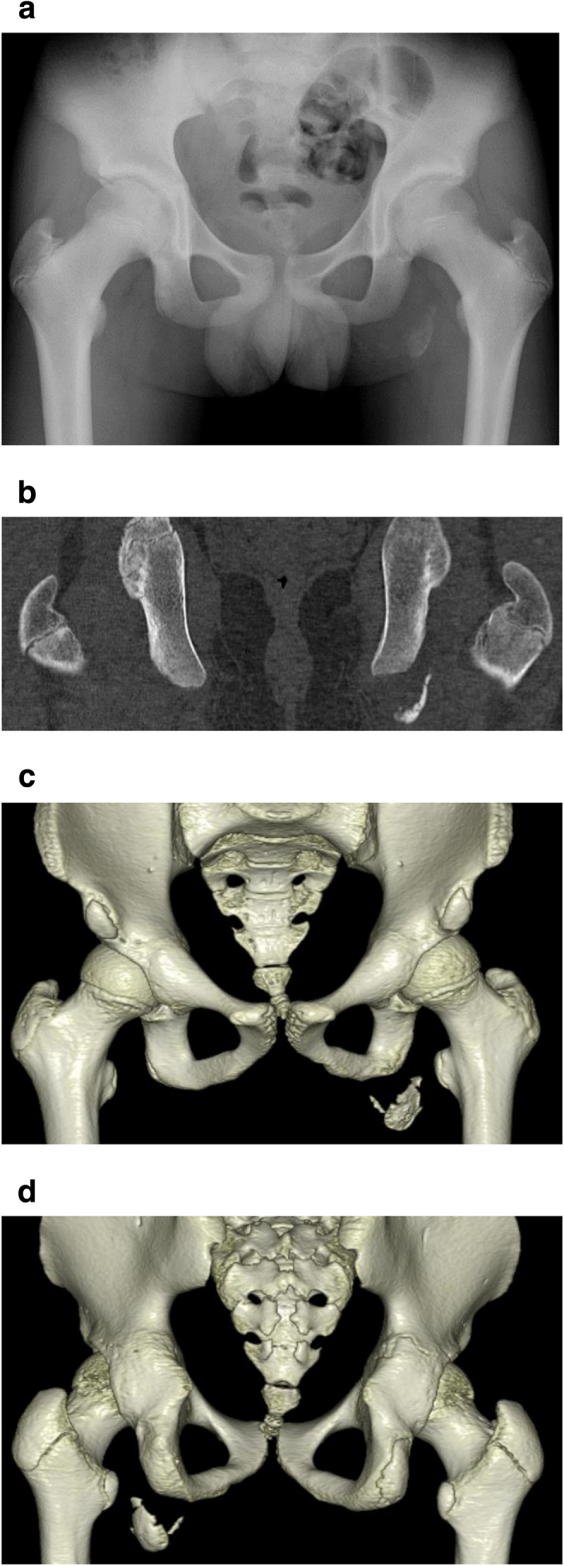
**a** Preoperative radiography reveals left ischial epiphyseal fracture. **b** CT shows 20 mm of displacement. **c** Anteroposterior preoperative three-dimensional CT (3D-CT). **d** Posteroanterior preoperative 3D-CT

**Fig. 2:**
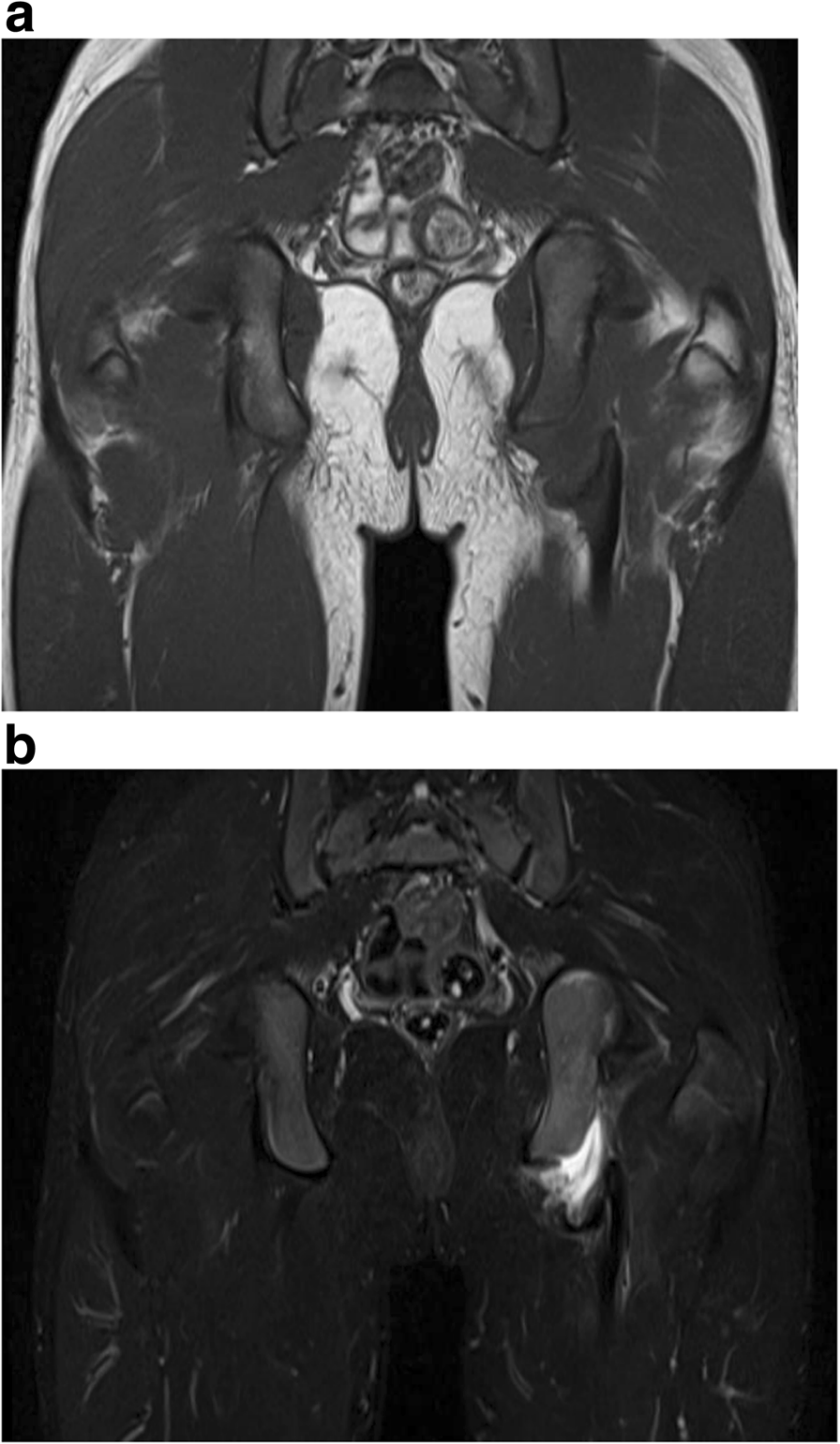
MRI two months postinjury reveals effusion at the area of epiphysiolysis and shows that the muscles have not been directly injured. **a** T1-weighted MRI. **b** T2-weighted short TI inversion recovery (STIR)

A 12-year-old boy presented with a left avulsion fracture of the ischial tuberosity. Informed consent was obtained from this patient and his family. The patient’s family history and previous medical history were unremarkable. The patient was a track-and-field athlete who felt severe pain in his left buttock while running. He visited a local hospital, where plain radiographs and computed tomography (CT) of the pelvis showed an avulsion fracture of the left ischium (Fig. 1). The fragment was displaced 20 mm. No neurological deficit was present. Complete non-weight-bearing therapy was performed as a conservative treatment, but the patient’s symptoms continued, and he visited our hospital two months after injury. During the preoperative assessment, he complained of pain in the gluteal area during walking. The patient also described muscle weakness of the hamstrings, and straight leg raising (SLR) was limited to 80°/60°. The results of a blood test were all within normal ranges. Magnetic resonance imaging (MRI) at two months postinjury revealed a displacement of approximately 20 mm, with fluid accumulation between the avulsed fragments (Fig. 2).

**Fig. 3:**
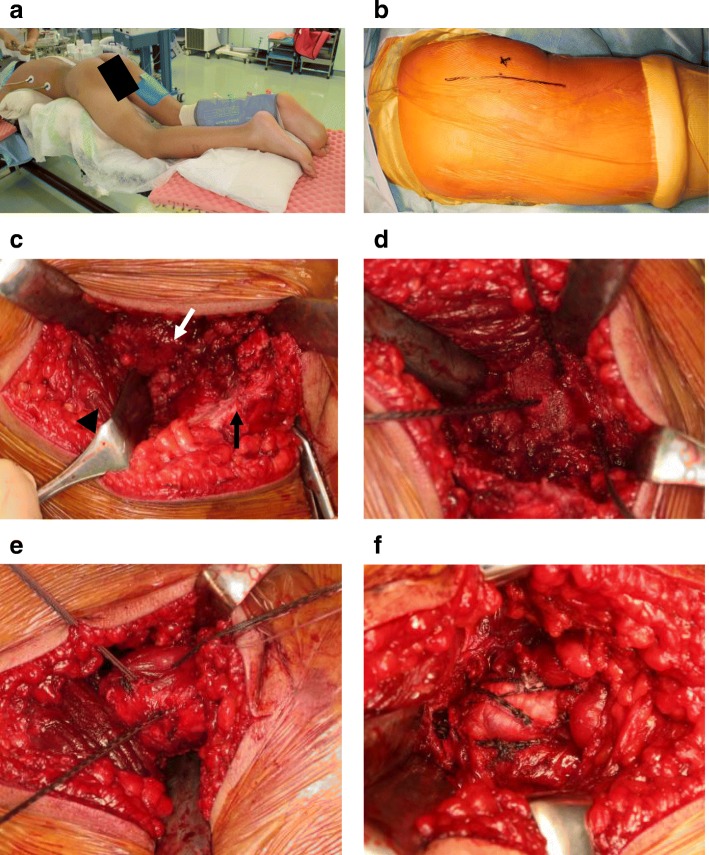
**a** Surgery is performed with the patient in the prone position, with hips and knees slightly flexed. **b** A longitudinal skin incision is made around the ischial tuberosity. **c** A subgluteal approach from the inferior margin of the gluteus maximus (arrow head) is used to reach the fracture segments, exposing the ischial tuberosity (white arrow) and the displaced fragment (black arrow). **d** Three anchor holes are made in the area of epiphysiolysis. **e**, **f** The fragment is reduced (**e**) then fixed using the suture bridge technique (**f**)

At eight weeks postinjury, we performed open reduction and anchor fixation because of non-union and displacement of the fragment after conservative therapy (Fig. 3). Following administration of general anesthesia, the patient was placed in a prone position. A 10-cm incision was made longitudinally around the ischial tuberosity, and subgluteal approach was used. The plane between the gluteus maximus and the hamstring muscles were divided. The inferior edge of the gluteus maximus was elevated to identify the ischial tuberosity. The avulsed fragment was distally displaced. The hamstrings were fully mobilized distally to reduce the avulsed fragment without excessive strain. Three suture anchors were placed in the exposed ischium (Fig. 3d). Two holes were drilled 1 cm distal to the proximal edge of the fragment, each in line with the distal suture anchors. Three drill holes were made through the avulsed fragment, taking into account the anchor locations. The fragment was reduced with the hip extended and the knee flexed and fixed with five biodegradable suture anchors (HEALIX ADVANCE 5.5; DePuy Synthes, Tokyo, Japan) using the suture bridge technique (Fig. 3e, f).

**Fig. 4:**
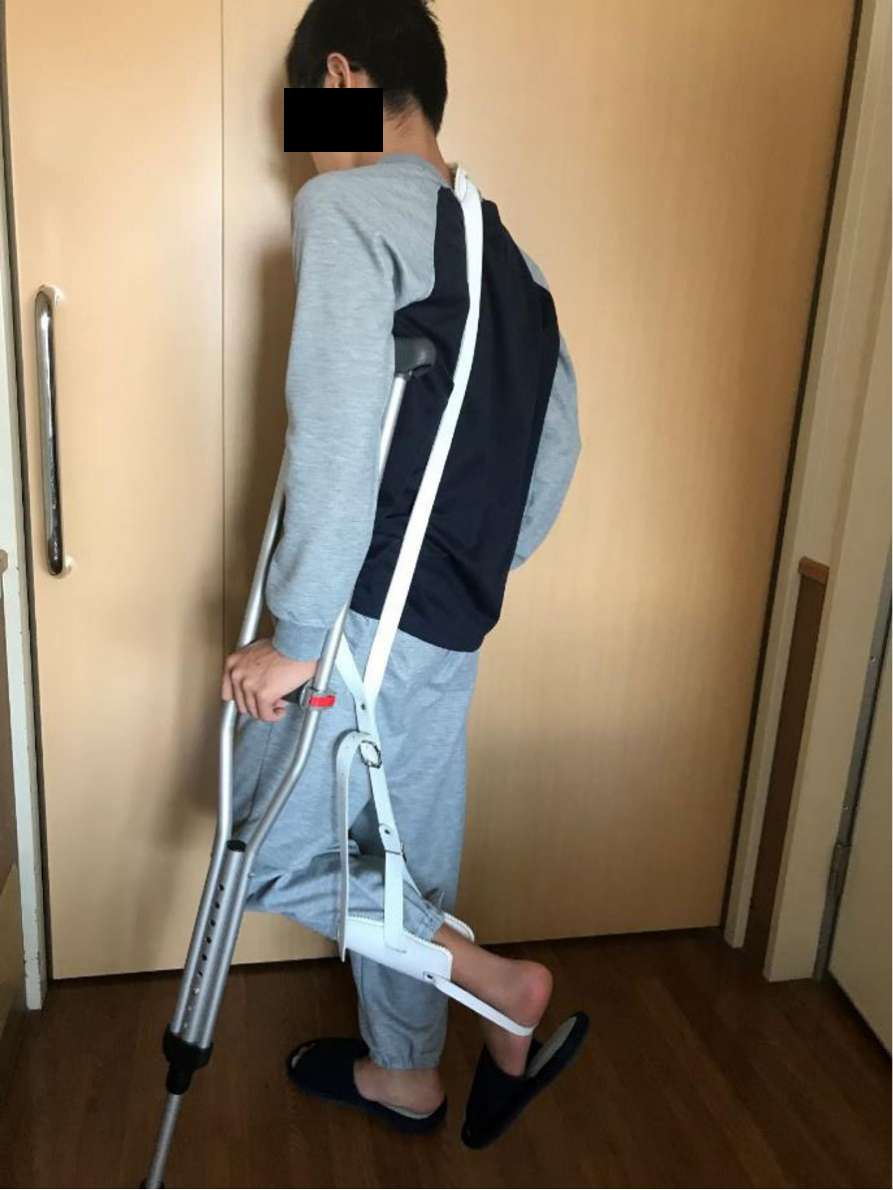
The Snyder sling is used postoperatively to limit knee extension

**Fig. 5:**
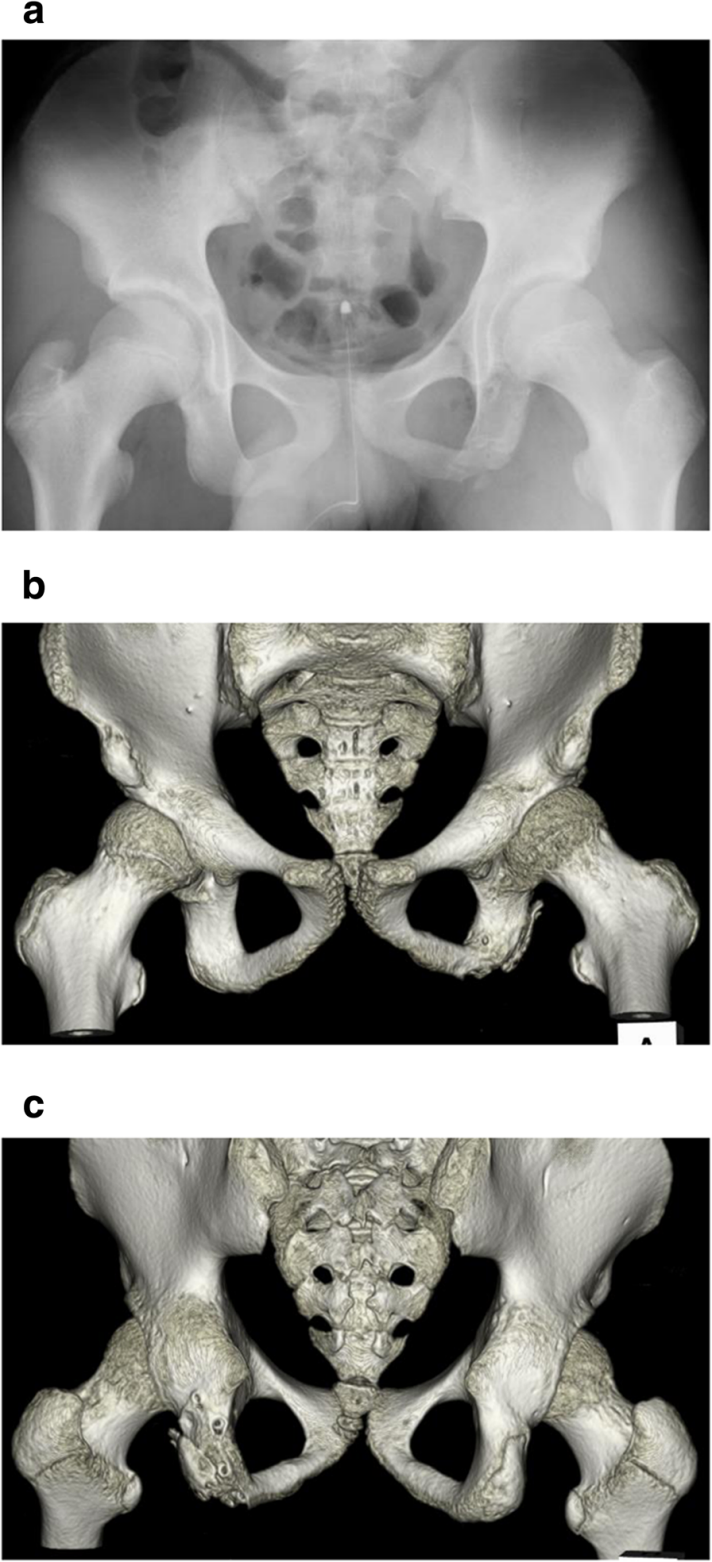
Postoperative imaging of the pelvis. **a** Plain radiography. **b**, **c** Postoperative CT

A Snyder sling (Hashimoto Artificial Limb Manufacture Co., Okayama, Japan) was used to restrict knee movement to within 45° of flexion in postoperative week 3 and to within 10° of flexion in week 5, while passive assistive hamstring stretches were performed; then, active ROM exercises were started from week 6 (Fig. 4). The next day after surgery, he started non-weight-bearing walking with crutches. One-third weight-bearing was permitted from week 6, and full weight-bearing was permitted from week 8. Union was confirmed on radiography and CT in week 9 (Fig. 5), and the patient was therefore permitted to start jogging and gradually building up training with squats and jumps involving quick hamstring stretching. The patient returned to competitive athletics in week 13. At the final follow-up, no bilateral difference was evident in hip ROM, at 120°/120° flexion or 30°/30° internal rotation. The SLR test was 80°/80°, and no pain was experienced in the ischial tuberosity during jogging. The manual muscle testing (MMT) score was 5 for both the gluteus maximus and hamstring muscles. Assessment when the patient returned to competition found no restriction of hip joint ROM, and his visual analog pain score was zero. The Lower Extremity Functional Scale (LEFS) [2] at the final follow-up was 80 points (100%).

## Discussion and conclusions

Since the first description by Berry in 1912 [3], avulsion fracture of the ischial tuberosity before epiphyseal arrest has been reported [1, 4, 5]. The mechanically vulnerable unfused apophysis can be injured as a result of traction force imposed by intense muscle contractions of the hamstrings during sports activities. This injury occurs most commonly during hurdles and high jump, possibly because of the eccentric contraction of the hamstrings when the leg is forced into hyperflexion of the hip with the knee fully extended [6–8].

Conservative treatment is the standard primary treatment modality for avulsion fracture of the ischial tuberosity [9, 10], but nonunion, fibrosis, overgrowth, buttock pain, and muscle weakness tend to occur [5, 11–13]. Avoca and Okay therefore reported that although patients with < 20 mm displacement might be successfully treated with conservative methods [14], surgical treatment is recommended if the displacement is ≥20 mm [15, 16]. Surgical treatment is also recommended for patients with sciatic nerve complications [17].

Wood et al. reported that a delay in surgical repair renders the repair more technically challenging, may increase the likelihood of sciatic nerve involvement, increases the need for postoperative bracing, and reduces postoperative outcomes in terms of hamstring strength and endurance [7]. In this case, we used a longitudinal incision. One disadvantage of the longitudinal incision is that this incision is cosmetically inferior to a transverse incision along the gluteal crease [4, 18]. However, the longitudinal incision enabled extension of the skin incision on demand, providing a good view for mobilization of the hamstrings. In addition, we did not expose the sciatic nerve in this case, avoiding the potential risk of sciatic nerve disturbance [8].

The surgical techniques reported previously include the use of a reconstruction plate, lag screws, and suture anchors [4, 19–21]. Kaneyama et al. reported the use of fixation with a cancellous screw and washer assembly [4]. Watts et al. attempted a minimally invasive surgical procedure involving percutaneous fixation using two cannulated cancellous screws but failed to reduce the fracture adequately [19]. Surgical techniques using suture anchors have recently been reported [21–23]. Biedert et al. reported the use of single-row suture anchor fixation in patients with displacement ≥20 mm, with a good final outcome [22]. However, they also reported that one patient needed operative revision one day after primary repair because of suture loosening. We consider that irrespective of whether screw fixation or suture anchor fixation is used, the shell-shaped avulsed fragment is difficult to fix using only one or two devices. An in vitro biomechanical analysis by Hamming et al. found that fixation with two anchors was mechanically insufficient and recommended fixing the avulsed fragment with five anchors [23]. This report appears to represent the first description of using the suture bridge technique with five suture anchors to treat avulsion fracture of the ischial tuberosity. The suture bridge technique is a rotator cuff repair technique that was first described by Park et al. in 2007 [24], with improved pressurized contact between the tendon and tuberosity compared with the double-row technique. In the present case, fixation using the suture bridge technique enabled stronger pressure between bone fragments over a wider area than that provided by simple suture anchor fixation.

The postoperative orthosis used in this case was a Snyder sling, a sling originally used to treat Perthes disease [25]. The Snyder sling is a variable angle brace, and we extended the knee in accordance with the state of hamstring stretching. We were thus able to gradually increase stretching stress on the hamstrings as bone union was achieved, eventually enabling a smooth return to sports activities. Skaara et al. reported that minor pain and limitations to activities of daily living were observed after surgical repair using the suture anchor technique, that isokinetic hamstring strength in the operated leg was significantly lower than that in the nonoperated leg and that a majority of patients did not trust the operated leg completely during physical activity [21]. In the present case in which the patient was treated with the suture bridge technique, the LEFS at the final follow-up was 100%. Although reports of surgical techniques and postoperative physical therapy for avulsion fracture of the ischial tuberosity are rare, good results may be achieved by combining good surgical therapy with aggressive physical therapy interventions to reduce stretching stress on the ischial tuberosity.

In conclusion, although the majority of avulsion fractures of the ischial tuberosity can be treated conservatively, patients with excessive displacement require surgical treatment. The suture bridge technique is a useful technique that provides sufficient strength for avulsion fracture of the ischial tuberosity.

## Abbreviations

CT: Computed tomography
MMT: Manual muscle testing
MRI: Magnetic resonance imaging
ROM: Range of motion
SLR: Straight leg raising
